# Psychological Treatment Effects Unrelated to Hair-Cortisol and Hair-BDNF Levels in Chronic Tinnitus

**DOI:** 10.3389/fpsyt.2022.764368

**Published:** 2022-02-18

**Authors:** Laura Basso, Benjamin Boecking, Patrick Neff, Petra Brueggemann, Birgit Mazurek, Eva M. J. Peters

**Affiliations:** ^1^Tinnitus Center, Charité – Universitätsmedizin Berlin, Berlin, Germany; ^2^Department of Psychiatry and Psychotherapy, University of Regensburg, Regensburg, Germany; ^3^University Research Priority Program “Dynamics of Healthy Aging”, University of Zurich, Zurich, Switzerland; ^4^Centre for Cognitive Neuroscience and Department of Psychology, University of Salzburg, Salzburg, Austria; ^5^Psychoneuroimmunology Laboratory, Department of Psychosomatic Medicine and Psychotherapy, Justus-Liebig University Giessen, Giessen, Germany; ^6^Psychosomatics and Psychotherapy, Charité Center 12 Internal Medicine and Dermatology, Charité – Universitätsmedizin Berlin, Berlin, Germany

**Keywords:** chronic tinnitus, stress, treatment, cognitive behavioral therapy (CBT), biomarker, cortisol, brain-derived neurotrophic factor (BDNF)

## Abstract

**Background:**

Currently, there are no objective markers to measure treatment efficacy in chronic (distressing) tinnitus. This study explores whether stress-related biomarkers cortisol and brain-derived neurotrophic factor (BDNF) measured in hair samples of chronic tinnitus patients change after compact multimodal tinnitus-specific cognitive behavioral therapy.

**Methods:**

In this longitudinal study, hair-cortisol and hair-BDNF levels, self-reported tinnitus-related distress (Tinnitus Questionnaire; TQ), and perceived stress (Perceived Stress Questionnaire; PSQ-20) were assessed before and 3 months after 5 days of treatment in *N* = 80 chronic tinnitus patients. Linear mixed-effects models with backward elimination were used to assess treatment-induced changes, and a cross-lagged panel model (structural equation model) was used for additional exploratory analysis of the temporal associations between TQ and hair-BDNF.

**Results:**

At follow-up, a reduction in TQ (*p* < 0.001) and PSQ-20 scores (*p* = 0.045) was observed, which was not influenced by baseline hair-cortisol or hair-BDNF levels. No changes in biomarker levels were observed after treatment. The exploratory analysis tentatively suggests that a directional effect of baseline TQ scores on hair-BDNF levels at follow-up (trend; *p* = 0.070) was more likely than the opposite directional effect of baseline hair-BDNF levels on TQ scores at follow-up (n.s.).

**Discussion:**

While the treatment effectively reduced tinnitus-related distress and perceived stress in chronic tinnitus patients, this effect was not mirrored in biological changes. However, the lack of changes in hair-cortisol and hair-BDNF levels might have been influenced by the treatment duration, follow-up interval, or confounding medical factors, and therefore must be interpreted with caution. The relationship between tinnitus-related distress and hair-BDNF levels should be explored further to obtain a better understanding of stress-related effects in chronic tinnitus.

## Introduction

Tinnitus is the subjective perception of a sound in absence of an external source. Chronic tinnitus is a frequent phenomenon with prevalence estimates in adults ranging up to 15% (1). In many affected individuals, tinnitus leads to considerable distress; constituting a big or very big problem for 7% and a moderate problem for 20% (2). Tinnitus associated with suffering can be conceptualized as “tinnitus disorder” (3) and is known to be influenced by personal vulnerability-stress interactions (4).

Currently, no existing treatment option can eliminate the tinnitus percept. However, the negative impact of tinnitus on the quality of life (QoL) in tinnitus patients can be reduced by cognitive behavioral therapy (5, 6). Cognitive behavioral therapy is a widely studied, evidence-based therapeutic approach that can be used for the treatment of various mental health problems (7). In the clinical care of tinnitus patients, cognitive behavioral therapy is focused on addressing dysfunctional cognitions, behaviors, and emotions related to tinnitus (which negatively affect the QoL) through cognitive restructuring and behavioral modification (5, 8). Because of the complex and multifactorial etiology and maintenance of chronic tinnitus, cognitive behavioral therapy-based multidisciplinary treatment approaches are recommended (9, 10). Multidisciplinary interventions for chronic tinnitus with cognitive behavioral therapy elements were found to be effective and have stable long-term effects (11–14).

At present, treatment efficacy can only be assessed by subjective measures; commonly, psychometric questionnaires are used (15, 16). Objective measures of treatment efficacy, e.g., biomarkers that are sensitive to distress-related treatment responses in individuals suffering from chronic tinnitus, would be highly useful, as they could provide objective criteria for the evaluation and comparison of treatment approaches.

Stress-related biomarkers such as cortisol are traditionally mainly quantified in biological fluids (saliva, blood, or urine) but can also be measured in hair. Hair sampling has the advantage of being non-invasive, less influenced by situational factors, and allowing direct measurement of long-term concentrations (cumulative concentrations over one or several months) without requiring repeated sampling (17).

Hair-cortisol is an established stress-related measure of cumulative cortisol secretion (18). However, the results of individual studies on its association with self-reported levels of perceived stress are not always conclusive (18).

Brain-derived neurotrophic factor (BDNF) is another stress-related marker that can be measured in hair (19). Among the important functions of BDNF is its involvement in neuroprotection and synaptic plasticity (20). Animal research has shown that BDNF expression is strongly affected by stress (21, 22). Moreover, peripheral BDNF levels appear to be decreased in stress-related mood disorders (23–25) and reduced BDNF expression may be involved in their pathogenesis (22). Peripheral BDNF levels have been shown to increase after antidepressant treatment in patients with major depressive disorder (23, 24, 26) and after mindfulness-based interventions in different study populations (27).

We previously investigated cross-sectional relationships between tinnitus loudness and distress with hair-cortisol and hair-BDNF in a sample of chronic tinnitus patients and observed a negative association between tinnitus-related distress and hair-BDNF (28), suggesting that hair-BDNF might be treatment-sensitive to psychological interventions in chronic tinnitus. The objective of the present longitudinal analysis of the same sample is to investigate treatment-induced changes in hair-cortisol and hair-BDNF levels to explore, for the first time, their potential as biomarkers of treatment efficacy.

This study has four research questions. (1) Whether tinnitus-related distress and perceived stress are reduced after compact multimodal tinnitus-specific cognitive behavioral therapy; which we expect to find based on previous studies that used a similar treatment approach (11–14). (2) Whether hair-cortisol or hair-BDNF levels show measurable and meaningful changes after the intervention. Based on our previous cross-sectional findings (28), suggesting that hair-cortisol is relatively independent of psychological factors in chronic tinnitus, no directional hypothesis was specified for hair-cortisol. However, based on the observed association with tinnitus-related distress, we expect hair-BDNF levels to increase in parallel with treatment-induced reductions in tinnitus-related distress. (3) Furthermore, we aim to identify which factors (sociodemographic, psychological, biological, tinnitus-/hearing-related, lifestyle, or hair-related) influence the outcome variables and respective treatment effects (questions 1 and 2). Linear mixed-effects models with backward elimination for each outcome will be used to address these research questions. (4) Based on the obtained results, an additional exploratory research question is to further investigate the temporal relationships between identified associated psychological and biological factors. Cross-lagged panel analysis will be used to assess such temporal relationships, accounting for the stability of the investigated factors over time.

## Materials and Methods

### Study Design and Sample

Between December 2018 and March 2020, 94 chronic tinnitus in-patients volunteered to participate in this study, which consisted of three measurements: (1) before and (2) directly after 5 days of compact multimodal tinnitus-specific cognitive behavioral therapy, which is the current standard clinical treatment for chronic tinnitus offered at the Tinnitus Center of Charité – Universitätsmedizin Berlin, and (3) a 3-month follow-up measurement (lasting until June 2020). Baseline data of the present study (*N* = 91 for hair-cortisol, *N* = 87 for hair-BDNF) have been previously analyzed in cross-section (28).

**Figure 1:**
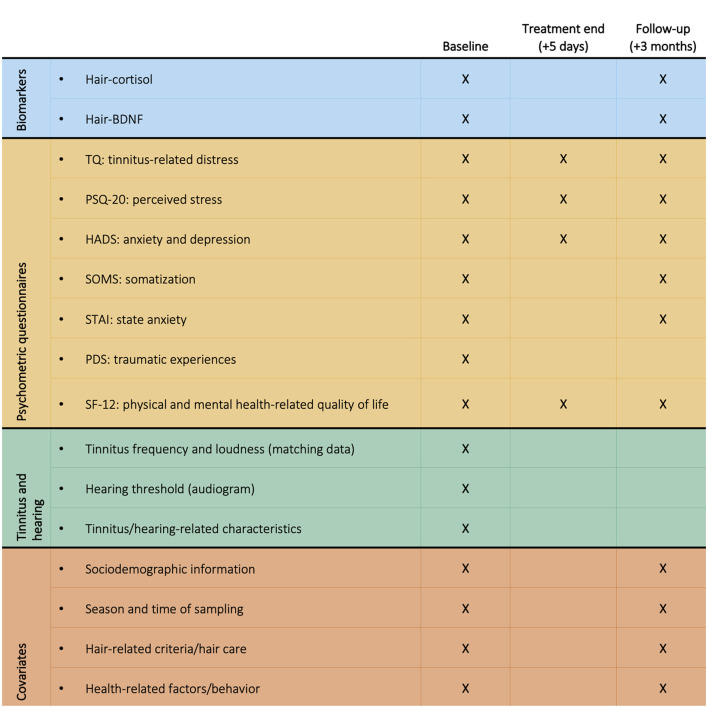
Overview of all collected study variables across measurements (baseline, treatment end, and follow-up). BDNF, Brain-Derived Neurotrophic Factor; HADS, Hospital Anxiety and Depression Scale; PDS, Posttraumatic Diagnostic Scale; PSQ-20, Perceived Stress Questionnaire (20 item version); SF-12, Short Form-12 Health Survey; SOMS, Screening of Somatoform Disorders; STAI, State-Trait Anxiety Inventory (State Anxiety); TQ, Tinnitus Questionnaire.

The baseline measurement included the collection of hair samples and psychometric questionnaires (day of treatment begin); additionally, pure tone audiograms and tinnitus matching data were collected from audiometric records (most recent measurement before treatment begin; M = 73.8 days prior, SD = 59.41). The second measurement, performed approx. 5 days later (directly after treatment end), only included psychometric questionnaires. The third measurement performed approx. 3 months later (M = 93.81 days, SD = 11.94), included hair sample collection and psychometric questionnaires. All collected variables are summarized in Figure 1. Primary outcomes were tinnitus-related distress, perceived stress, hair-cortisol, and hair-BDNF.

Inclusion criteria were “diagnosis of chronic subjective tinnitus”, “age ≥ 18 years”, and “written informed consent” (28). Exclusion criteria were “inability to consent due to serious mental or physical impairments”, “simultaneous participation in other research studies”, “hair length <3 cm”, “any chemical hair treatment within 1 month prior to sampling (dying, bleaching, perming, or else)”, “hair washing or the use of hair products (hair mousse, hair gel, hair wax, hair spray) within 3 days prior to sampling”, and “hair combing on the day of sampling” (28).

One patient was excluded due to missing questionnaire data at baseline, three patients were excluded due to hair-related criteria (at baseline or follow-up), four patients did not complete the follow-up measurement (due to the associated effort), and six patients were excluded due to incomplete biomarker measures. The final sample size was *N* = 80. The remaining missing values, mostly of tinnitus matching data, were imputed (see Section Linear Mixed-Effects Models).

All participants were European; on average, 50.96 years old (SD = 11.72), and 66.25% (*N* = 53) were female. The study was approved by the local ethic commission of Charité – Universitätsmedizin Berlin (No. EA1/035/16) and all participants provided written informed consent.

### Compact Multimodal Tinnitus-Specific Cognitive Behavioral Therapy

The treatment took place over 4.78 days on average (SD = 1.10, range: 4–9), had a tinnitus-specific cognitive behavioral therapy focus (individual and group treatment sessions), and included the following other modalities: education, counseling, otorhinolaryngological and general medical diagnostics, auditory attention training, relaxation, and physiotherapeutic sessions.

### Psychometric Questionnaires and Covariates

The following psychometric questionnaires were used (German versions):

- *Tinnitus Questionnaire* (TQ) (29).
- *Perceived Stress Questionnaire* (PSQ-20; 20 item version) (30, 31).
- *Hospital Anxiety and Depression Scale* (HADS) (32, 33).
- *Screening of Somatoform Disorders* (SOMS; 7 days version) (34).
- *State-Trait Anxiety Inventory (STAI) – State Anxiety* (35).
- *Posttraumatic Diagnostic Scale (PDS) – Event List* (36).
- *Short Form-12 Health Survey* (SF-12; version 2) (37, 38).

Covariates included sociodemographic information, information on hair care, and health-related behavior [see (28)].

### Audiometry (Hearing Threshold and Tinnitus Pitch and Loudness Matching)

The mean hearing threshold at the frequencies 0.25, 0.5, 1, 2, 3, 4, 6, and 8 kHz measured by pure tone audiogram was calculated and averaged across ears if possible. The matched tinnitus frequency (Hz) and absolute loudness (dB) were averaged for bilateral tinnitus. Tinnitus pitch and loudness matching could not be performed in 21 patients [see (28)].

### Hair Sampling

Hair samples were cut with scissors from the region of the posterior vertex, as close to the scalp as possible. The median sampling time was 09:55 a.m. at baseline and 10:15 a.m. at follow-up. Samples were stored (in a dark container at room temperature) until analysis in summer/autumn 2020. The most proximal 1-cm hair segment was analyzed, one month prior to sampling. Cortisol and BDNF quantification was performed using commercial kits and followed the previously described laboratory protocol (19). According to the manufacturer, the sensitivity of the cortisol ELISA is 0.005 μg/dl (standard range 0.15–30 ng/ml) and of the BDNF ELISA 15.6 pg/ml (standard range 0–1000 pg/ml; BDNF measurements were performed in a dilution of 1:1000). The intra- and inter-assay coefficients of variation as stated by the manufacturer are +4.3 and +13.2% for cortisol ELISA, and +3.7 and +8.5% for BDNF ELISA, respectively. In our study, the intra- and inter-assay coefficients of variation were +1.91% and 7.49 ± 2.81 for cortisol, and +2.73% and 5.31 ± 3.35 for BDNF. All but seven BDNF values were within the detection range.

### Statistical Analysis

Analyses were performed using R (version 4.0.0) (39). Hair-cortisol values were log-transformed to establish normal distribution. For descriptive analyses, biomarker values between participants using antidepressant medication and the rest of the sample were compared using two-sample *t-*tests. To address research questions 1–3, linear mixed-effects models were calculated for TQ, PSQ-20, hair-cortisol, and hair-BDNF as outcome variables, and these models were reduced by backward elimination to identify relevant predictors. For research question 4, cross-lagged panel analysis was used. The following packages were used for linear mixed-effects models: “lme4” for model building; “lmerTest” for backward elimination; “multcomp” for significance testing; “MuMIn” for estimates of marginal and conditional R^2^, “sjPlot” for fixed effects plots; “glmmTMB” for diagnostic plots. For imputation of missing values “DMwR2” was used and for the cross-lagged panel analysis the packages “lavaan” and “semPlot”. The significance threshold was set to *p* < 0.05.

#### Linear Mixed-Effects Models

Numeric predictors were centered and scaled. Missing values were imputed with k-nearest neighbor imputation (see below).

##### Model Building and Selection

First, the “full” model was estimated including all predictors of interest and their respective interaction terms with “measurement” (baseline, treatment end, and follow-up for psychometric questionnaires; baseline and follow-up for biomarkers) as fixed effects. For TQ and PSQ-20, selected predictors included sociodemographic factors, tinnitus- and hearing-related factors, psychometric factors, and biomarker scores at baseline, as well as interaction terms of all these baseline factors with the measurement variable. For hair-cortisol and hair-BDNF, selected predictors included sociodemographic factors, tinnitus- and hearing-related factors, tinnitus matching (loudness/frequency), psychometric factors and covariates, either at baseline or both measurements, as well as interaction terms of all baseline factors with the measurement variable; for time-varying covariates, no interaction terms were included. Covariates for the biomarker models were selected based on cross-sectional results (28).

Second, the random-effects structure was determined by comparing random intercept models with random intercept and slope models. For the prediction of TQ scores, no significant difference was present between the random intercept and random intercept and slope models, $\chi_{\left(2\right)}^{2}=$ 1.20, *p* = 0.549. For the other outcomes, the comparison was not possible due to singular fit (PSQ-20) or an insufficient number of observations (hair-cortisol and hair-BDNF) for estimation of the respective random intercept and slope models. Consequently, for all outcomes, the more parsimonious random intercept model was chosen. Lastly, automated backward elimination was performed to obtain the final reduced model.

**Figure 2:**
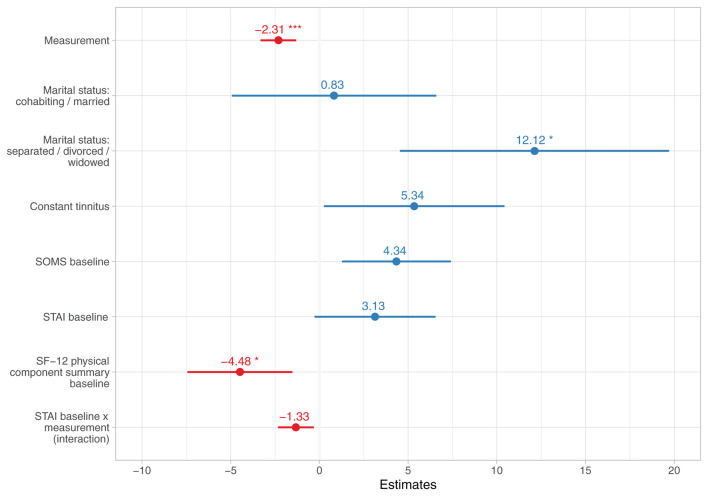
Reduced linear mixed-effects model with stepwise backward elimination for change in Tinnitus Questionnaire (TQ) scores across baseline, treatment end, and follow-up (*N* = 80). Numbers indicate estimated coefficient effects and lines depict 95% confidence intervals. Significance levels are displayed after adjustment for multiple testing with Holm's method. SF-12, Short Form-12 Health Survey; SOMS, Screening of Somatoform Disorders; STAI, State-Trait Anxiety Inventory (State Anxiety). *p < 0.05; ***p < 0.001.

**Figure 3:**
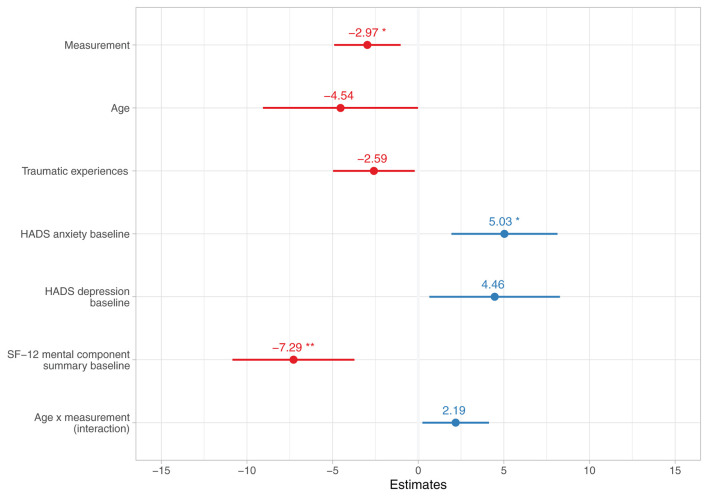
Reduced linear mixed-effects model with stepwise backward elimination for change in Perceived Stress Questionnaire (PSQ-20) scores across baseline, treatment end, and follow-up (*N* = 80). Numbers indicate estimated coefficient effects and lines depict 95% confidence intervals. Significance levels are displayed after adjustment for multiple testing with Holm's method. HADS, Hospital Anxiety and Depression Scale, SF-12, Short Form-12 Health Survey. *p < 0.05; **p < 0.01.

**Figure 4:**
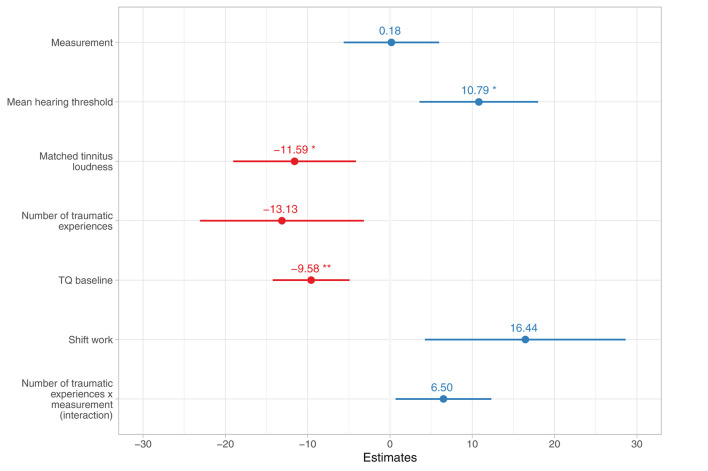
Reduced linear mixed-effects model with stepwise backward elimination (*N* = 80) for change in hair-BDNF levels across baseline and follow-up (*N* = 80). Numbers indicate estimated coefficient effects and lines depict 95% confidence intervals. Significance levels are displayed after adjustment for multiple testing with Holm's method. TQ, Tinnitus Questionnaire. *p < 0.05; **p < 0.01.

Models were fitted using restricted maximum likelihood (REML) (40). For significance testing, *z-*tests were used (41); *p*-values were adjusted for multiple testing (see below). Model equations, model fit, fixed effects estimates with 95% confidence intervals, and random effect variance of the full and reduced models for each outcome can be found in Tables 3–6. Fixed effects estimates with 95% confidence intervals of the reduced models are displayed in Figures 2–4 and test statistics of significant effects after adjustment are reported. Diagnostic plots for each outcome can be found in the Supplementary Figures 1–4.

##### Imputation

Imputation of missing values was performed before model building using k-nearest neighbor imputation. The following missing values were imputed: *N* = 21 for tinnitus loudness and frequency, *N* = 5 for hair color, *N* = 3 for SF-12, and *N* = 1 for hearing aid use. The high correlation of tinnitus loudness with mean hearing threshold was preserved after imputation (without imputation: Spearman *r* = 0.798, *p* < 0.001, with imputation: *r* = 0.803, *p* < 0.001), see Supplementary Figure 5.

##### Adjustment for Multiple Testing

All *p*-values of the fixed effects of all four reduced models (28 effects in total) were adjusted for multiple testing using Holm's method (42) (using “p.adjust”), as this method is more powerful than Bonferroni correction (43).

#### Exploratory Analysis: Cross-Lagged Panel Model

As an exploratory analysis (research question 4) based on the obtained results, a cross-lagged panel model was calculated to investigate temporal relations between tinnitus-related distress and hair-BDNF levels in structural equation modeling framework using maximum likelihood estimation with robust standard errors. Previously identified influencing factors on TQ and BDNF levels from the reduced linear mixed-effects models were included as control variables. Standardized estimates (based on latent variable variance), standard errors, and *p*-values are reported. Due to the exploratory nature of this analysis, *p*-values were not adjusted.

## Results

### Sample Description

Sample characteristics across measurements are summarized in Table 1 (numeric variables) and Table 2 (categorical variables). Musculoskeletal symptoms like muscular imbalance (*N* = 46, 58.23%), segmental joint dysfunction (*N* = 46, 58.23%), chronic cervical syndrome (*N* = 44, 55.70%), craniomandibular/temporomandibular dysfunction (*N* = 31, 39.24%), and bruxism (*N* = 35, 44.30%), were common comorbidities in the sample (*N* = 79). None of the participants suffered from endocrine conditions with altered cortisol production (Cushing syndrome or Addison's disease) or from neurodegenerative diseases associated with changes in cortisol and BDNF levels like Alzheimer's disease, Parkinson's disease, or Huntington's disease (44, 45). Past substance abuse was reported by *N* = 2 participants (2.53%). Eleven patients (14.10%) were using antidepressants; their baseline hair-BDNF (M = 69.01, SD = 27.93 vs. M = 79.61, SD = 28.40; *t*_(76)_ = −1.15, *p* = 0.254) and (log-transformed) hair-cortisol values (M = −1.23, SD = 0.27 vs. M = −1.44, SD = 0.35; *t*_(76)_ = 1.93, *p* = 0.057) did not significantly differ from the rest of the sample (*N* = 67).

**Table 1:** Summary statistics of numeric variables (*N* = 80).

	**Baseline**				**Treatment end**				**Follow-up**			
**Variable**	**Mean**	**SD**	**Min**	**Max**	**Mean**	**SD**	**Min**	**Max**	**Mean**	**SD**	**Min**	**Max**
TQ total score: tinnitus-related distress	34.70	15.61	3	79	31.88	15.11	2	75	30.08	15.74	0	79
PSQ-20 total score: perceived stress	51.79	19.10	8.33	86.67	41.48	21.07	0	88.33	45.85	20.42	3.33	85
Cortisol μg/dl	0.052	0.042	0.004	0.211					0.046	0.045	0.004	0.288
BDNF ng/ml	78.35	28.08	12.62	130.03					78.53	28.89	16.40	130.13
Age	50.96	11.72	19	75								
Mean hearing threshold (dB)	21.64	12.77	4.69	71.56								
Matched tinnitus frequency (Hz) (*N =* 59)	5,491.53	2,422.49	250	10,000								
Matched tinnitus loudness (dB) (*N =* 59)	37.99	20.06	5	79								
HADS: anxiety	7.90	4.13	0	18	7.19	4.07	0	17	6.85	3.78	0	16
HADS: depression	5.97	3.94	0	14	5.42	3.87	0	13	6.16	4.19	0	16
SF-12: physical component summary (*N =* 79/78/80)	42.08	10.24	16.05	59.08	44.51	9.56	18.76	59.08	43.20	9.58	18.75	59.08
SF-12: mental component summary (*N =* 79/78/80)	37.75	10.63	16.05	57.53	43.70	10.03	19.16	59.22	39.57	9.85	17.63	57.53
SOMS: somatization	9.22	7.06	0	29					9.20	6.44	0	24
STAI total score: state anxiety	44.42	11.25	26	75					41.23	11.30	23	70
PDS: number of traumatic experiences	1.68	1.34	0	5								
Frequency of hair washing per week	2.91	1.66	1	8								
Alcohol units per week^a^	2.12	4.02	0	21					2.65	3.24	0	18
BMI	25.75	4.55	17.62	41.38					25.42	4.18	17.93	37.13
Physical activity score^b^	6.04	5.95	0	24					6.26	5.52	0	28
Cups of coffee/tea per day	2.84	1.90	0	9					2.60	1.86	0	8

a *Alcohol units consumed per week: one unit = 0.3 l beer or 0.2 l wine or shot glass of spirits*.

b *Physical activity score: number of days per week on which participants are physically active times the duration of the physical activity (1 = less than 10 min., 2 = 10–30 min., 3 = 30–60 min., 4 = more than 60 min). BDNF, Brain-Derived Neurotrophic Factor; BMI, Body Mass Index; HADS, Hospital Anxiety and Depression Scale; PDS, Posttraumatic Diagnostic Scale; PSQ-20, Perceived Stress Questionnaire (20 item version); SF-12, Short Form-12 Health Survey; SOMS, Screening of Somatoform Disorders; STAI, State-Trait Anxiety Inventory (State Anxiety); TQ, Tinnitus Questionnaire*.

**Table 2:** Summary statistics of categorical variables (*N* = 80).

	**Baseline**		**Follow-up**	
**Variable**	**N**	**%**	**N**	**%**
Sex: female	53	66.25		
Education level^a^				
Low	13	16.25		
Medium	29	36.25		
High	38	47.50		
Marital status				
Single	22	27.50	20	25
Cohabiting / married	44	55	45	56.25
Separated / divorced / widowed	14	17.50	15	18.75
Employment: yes	62	77.50	58	72.50
Tinnitus type				
Intermittent	49	61.25	43	53.75
Constant	31	38.75	37	46.25
Tinnitus onset associated with stress: yes	43	53.75		
Tinnitus influenced by stress: yes	64	80	77	96.25
Hyperacusis (self-report): yes	62	77.50	68	85
Use of hearing aids: yes	14	17.50	18	22.50
Missing			1	1.25
Season of sample collection				
Winter	40	50	11	13.75
Spring	12	15	40	50
Summer	18	22.50	15	18.75
Autumn	10	12.50	14	17.50
Regular use of hair products: yes	28	35		
Hair color				
Gray / white	15	18.75		
Blonde / red	27	33.75		
Brown / black	33	41.25		
I don't know / missing	5	6.25		
Smoking: yes	10	12.50	11	13.75
Shift work: yes	14	17.50		
Sport				
Less than 1 h a week	29	36.25	24	30
Regularly, 1–2 h a week	34	42.50	30	37.50
Regularly, 3–4 h a week	14	17.50	20	25
Regularly, more than 4 h a week	3	3.75	6	7.50

a *Education levels: low = elementary, secondary or middle school; medium = high school or completed apprenticeship; high = university*.

### Linear Mixed-Effects Models

#### Tinnitus Questionnaire (TQ): Reduction Across Baseline, Treatment End, and Follow-Up

To investigate the change in tinnitus-related distress as measured by the TQ (research question 1) and relevant modulating influences (research question 3), two linear mixed-effects models were calculated, the first including all potentially relevant factors (full model) and the second after excluding non-significant factors by backward elimination (reduced model); the results of both models can be found in Table 3. The following significant fixed effects estimates were identified in the reduced model after adjustment for multiple testing (see Figure 2): A reduction in TQ scores across measurements, β = −2.31 [−3.31, −1.31], *z* = −4.53, *p*_unadjusted_ < 0.001, *p*_adjusted_ < 0.001, generally higher TQ scores in separated, divorced, or widowed patients, β = 12.12 [4.58, 19.67], *z* = 3.15, *p*_unadjusted_ = 0.002, *p*_adjusted_ = 0.033, and generally lower TQ scores in patients with higher SF-12 physical component summary baseline scores, i.e., higher physical health-related QoL, β = −4.48 [−7.42, −1.53], *z* = −2.98, *p*_unadjusted_ = 0.003, *p*_adjusted_ = 0.049.

**Table 3:** Full and backward reduced linear mixed-effects models for change in Tinnitus Questionnaire (TQ) scores across baseline, treatment end, and follow-up (*N* = 80).

	**Full model**	**Backward reduced model**
	**Fixed effects estimates (95% confidence intervals)**	
**Measurement**	−2.58 (−6.85, 1.68)	−2.31^***^(−3.31, **−1.31)**
Sex: male (vs. female)	−1.17 (−9.30, 6.96)	
Age	−0.07 (−5.28, 5.14)	
Cohabiting / married (vs. single)	0.59 (−9.76, 10.95)	0.83 (−4.90, 6.56)
**Separated / divorced / widowed (vs. single)**	9.77 (−3.48, 23.02)	12.12^*^(4.58, 19.67)
Education: linear	−1.80 (−9.72, 6.13)	
Education: quadratic	−1.06 (−7.81, 5.69)	
Employment: no (vs. yes)	5.36 (−4.29, 15.02)	
Mean hearing threshold	−1.29 (−6.81, 4.23)	
Tinnitus onset associated with stress: yes (vs. no)	3.90 (−4.01, 11.80)	
Constant tinnitus (vs. intermittent)	7.11 (−0.64, 14.87)	5.34 (0.29, 10.40)
Tinnitus influenced by stress: yes (vs. no)	−1.50 (−11.83, 8.84)	
Hearing aids: yes (vs. no)	1.36 (−11.53, 14.24)	
Hyperacusis: yes (vs. no)	−6.08 (−16.48, 4.33)	
Number of traumatic experiences	−1.42 (−5.65, 2.82)	
SOMS baseline	1.05 (−4.17, 6.27)	4.34 (1.29, 7.40)
STAI baseline	2.19 (−3.86, 8.24)	3.13 (−0.26, 6.53)
PSQ-20 baseline	1.66 (−5.89, 9.21)	
HADS anxiety baseline	0.64 (−5.71, 7.00)	
HADS depression baseline	3.90 (−3.00, 10.79)	
**SF-12 physical component summary baseline**	−4.88 (−10.79, 1.03)	−4.48^*^(−7.42, **−1.53)**
SF-12 mental component summary baseline	2.99 (−4.78, 10.75)	
Hair-cortisol baseline	0.86 (−2.96, 4.69)	
Hair-BDNF baseline	−3.26 (−7.43, 0.91)	
Measurement × sex	0.20 (−2.39, 2.78)	
Measurement × age	0.79 (−0.87, 2.45)	
Measurement × cohabiting / married	−0.23 (−3.53, 3.06)	
Measurement × separated / divorced / widowed	0.25 (−3.96, 4.47)	
Measurement × education (linear)	−0.41 (−2.93, 2.11)	
Measurement × education (quadratic)	0.25 (−1.90, 2.39)	
Measurement × no employment	−1.28 (−4.35, 1.80)	
Measurement × mean hearing threshold	0.44 (−1.32, 2.19)	
Measurement × tinnitus onset associated with stress	−0.76 (−3.28, 1.75)	
Measurement × constant tinnitus	−0.83 (−3.30, 1.64)	
Measurement × tinnitus influenced by stress	−0.73 (−4.02, 2.56)	
Measurement × hearing aids	0.05 (−4.05, 4.15)	
Measurement × hyperacusis	2.55 (−0.76, 5.86)	
Measurement × number of traumatic experiences	0.36 (−0.99, 1.71)	
Measurement × SOMS baseline	1.05 (−0.61, 2.71)	
Measurement × STAI baseline	−1.46 (−3.39, 0.46)	−1.33 (−2.33, −0.32)
Measurement × PSQ-20 baseline	0.03 (−2.38, 2.43)	
Measurement × HADS anxiety baseline	−0.45 (−2.47, 1.58)	
Measurement × HADS depression baseline	−0.70 (−2.90, 1.49)	
Measurement × SF-12 physical component summary baseline	1.28 (−0.60, 3.16)	
Measurement × SF-12 mental component summary baseline	−1.49 (−3.96, 0.98)	
Measurement × hair-cortisol baseline	0.08 (−1.13, 1.30)	
Measurement × hair-BDNF Baseline	0.43 (−0.90, 1.75)	
Constant	35.17^***^ (21.77, 48.58)	32.19^***^ (27.06, 37.33)
	**Random effects variance (SD)**	
Subject (random intercept)	117.06 (10.82)	104.28 (10.21)
	**Model fit**	
Log-likelihood	−797.65	−858.22
Aikake information criterion	1,695.31	1,738.44
Bayesian information criterion	1,869.34	1,776.73
Marginal R^2^	0.43	0.42
Conditional R^2^	0.84	0.83

*Linear mixed model fit by REML; z-tests were used to test fixed effects estimates; significance levels are displayed after adjustment for multiple testing with Holm's method; significant effects in the reduced model are printed in bold. Observations = 240. **Model** **equations**: Full model: “TQ ~ measurement + sex × measurement + age × measurement + marital status × measurement + education × measurement + employment × measurement + mean hearing threshold × measurement + tinnitus onset associated with stress × measurement + tinnitus type × measurement + tinnitus influenced by stress × measurement + hearing aids × measurement + hyperacusis × measurement + number of traumatic experiences × measurement + SOMS baseline × measurement + STAI baseline × measurement + PSQ-20 baseline × measurement + HADS anxiety baseline × measurement + HADS depression baseline × measurement + SF-12 physical component summary baseline × measurement + SF-12 mental component summary baseline × measurement + hair-cortisol baseline × measurement + hair-BDNF baseline × measurement + (1 | subject)”. Reduced model: “TQ ~ measurement + marital status + tinnitus type + SOMS baseline + STAI baseline + SF-12 physical component summary baseline + STAI baseline × measurement + (1 | subject).” BDNF, Brain-Derived Neurotrophic Factor; HADS, Hospital Anxiety and Depression Scale; PDS, Posttraumatic Diagnostic Scale; PSQ-20, Perceived Stress Questionnaire (20 item version); SF-12, Short Form-12 Health Survey; SOMS, Screening of Somatoform Disorders; STAI, State-Trait Anxiety Inventory (State Anxiety); TQ, Tinnitus Questionnaire*.

* *p < 0.05*;

*** *p < 0.001*.

#### Perceived Stress Questionnaire (PSQ-20): Reduction Across Baseline, Treatment End, and Follow-Up

To investigate the change in perceived stress levels as measured by the PSQ-20 (research question 1) and relevant modulating influences (research question 3), two linear mixed-effects models were calculated, the first including all potentially relevant factors (full model) and the second after excluding non-significant factors by backward elimination (reduced model); the results of both models can be found in Table 4. The following significant fixed effects estimates were identified in the reduced model after adjustment for multiple testing (see Figure 3): A reduction in PSQ scores across measurements, β = −2.97 [−4.90, −1.04], *z* = −3.02, *p*_unadjusted_ = 0.003, *p*_adjusted_ = 0.045, generally higher PSQ scores in patients with higher HADS anxiety baseline scores, β = 5.03 [1.95, 8.11], *z* = 3.20, *p*_unadjusted_ = 0.001, *p*_adjusted_ = 0.028, and generally lower PSQ scores in patients with higher SF-12 mental component summary baseline scores, i.e., higher mental health-related QoL, β = −7.29 [−10.83, −3.75], *z* = −4.03, *p*_unadjusted_ < 0.001, *p*_adjusted_ = 0.001.

**Table 4:** Full and backward reduced linear mixed-effects models for change in Perceived Stress Questionnaire (PSQ-20) scores across baseline, treatment end, and follow-up (*N* = 80).

	**Full model**	**Backward reduced model**
	**Fixed effects estimates (95% confidence intervals)**	
**Measurement**	−2.76 (−10.94, 5.42)	−2.97^*^(−4.90, **−1.04)**
Sex: male (vs. female)	−5.01 (−16.24, 6.23)	
Age	−3.93 (−11.28, 3.41)	−4.54 (−9.05, −0.04)
Cohabiting / married (vs. single)	1.29 (−13.25, 15.84)	
Separated / divorced / widowed (vs. single)	−2.25 (−21.79, 17.29)	
Education: linear	−1.81 (−13.02, 9.41)	
Education: quadratic	−2.05 (−11.63, 7.54)	
Employment: no (vs. yes)	−4.20 (−18.02, 9.62)	
Mean hearing threshold	0.97 (−6.86, 8.80)	
Tinnitus onset associated with stress: yes (vs. no)	0.02 (−11.26, 11.30)	
Constant tinnitus (vs. intermittent)	5.00 (−6.29, 16.29)	
Tinnitus influenced by stress: yes (vs. no)	2.59 (−12.08, 17.25)	
Hearing aids: yes (vs. no)	−5.46 (−23.62, 12.71)	
Hyperacusis: yes (vs. no)	−1.60 (−16.33, 13.14)	
Number of traumatic experiences	−2.77 (−8.68, 3.14)	−2.59 (−4.96, −0.22)
SOMS baseline	−4.44 (−11.59, 2.72)	
STAI baseline	0.90 (−7.66, 9.46)	
TQ baseline	0.74 (−6.23, 7.72)	
**HADS anxiety baseline**	5.41 (−3.02, 13.83)	5.03^*^(1.95, 8.11)
HADS depression baseline	8.41 (−0.82, 17.65)	4.46 (0.67, 8.25)
SF-12 physical component summary baseline	−1.70 (−10.20, 6.81)	
**SF-12 mental component summary baseline**	−7.76 (−17.87, 2.35)	−7.29^**^(−10.83, **−3.75)**
Hair-cortisol baseline	−0.34 (−5.67, 4.99)	
Hair-BDNF baseline	0.48 (−5.63, 6.58)	
Measurement × sex	0.67 (−4.15, 5.50)	
Measurement × age	1.64 (−1.51, 4.79)	2.19 (0.26, 4.12)
Measurement × cohabiting / married	0.34 (−5.91, 6.59)	
Measurement × separated / divorced / widowed	−0.34 (−8.73, 8.05)	
Measurement × education (linear)	1.93 (−2.88, 6.75)	
Measurement × education (quadratic)	0.86 (−3.26, 4.97)	
Measurement × no employment	1.99 (−3.95, 7.92)	
Measurement × mean hearing threshold	−0.43 (−3.79, 2.93)	
Measurement × tinnitus onset associated with stress	0.91 (−3.93, 5.76)	
Measurement × constant tinnitus	−2.64 (−7.49, 2.21)	
Measurement × tinnitus influenced by stress	−4.17 (−10.47, 2.13)	
Measurement × hearing aids	2.16 (−5.64, 9.96)	
Measurement × hyperacusis	2.69 (−3.64, 9.02)	
Measurement × number of traumatic experiences	−0.03 (−2.57, 2.50)	
Measurement × SOMS baseline	1.46 (−1.62, 4.53)	
Measurement × STAI baseline	0.56 (−3.11, 4.24)	
Measurement × TQ baseline	1.14 (−1.86, 4.13)	
Measurement × HADS anxiety baseline	−1.19 (−4.80, 2.43)	
Measurement × HADS depression baseline	−2.19 (−6.15, 1.78)	
Measurement × SF-12 physical component summary baseline	1.11 (−2.54, 4.76)	
Measurement × SF-12 mental component summary baseline	−0.06 (−4.41, 4.28)	
Measurement × hair-cortisol baseline	−0.34 (−2.63, 1.94)	
Measurement × hair-BDNF baseline	−0.10 (−2.72, 2.52)	
Constant	53.13^***^ (34.09, 72.18)	52.31^***^ (47.84, 56.78)
	**Random effects variance (SD)**	
Subject (random intercept)	62.31 (7.89)	55.26 (7.43)
	**Model fit**	
Log-likelihood	−883.36	−961.61
Aikake information criterion	1,866.73	1,943.22
Bayesian information criterion	2,040.76	1,978.03
Marginal R^2^	0.52	0.51
Conditional R^2^	0.65	0.64

*Linear mixed model fit by REML; z-tests were used to test fixed effects estimates; significance levels are displayed after adjustment for multiple testing with Holm's method; significant effects in the reduced model are printed in bold. Observations = 240. **Model equations:** Full model: “PSQ-20 ~ measurement + sex × measurement + age × measurement + marital status × measurement + education × measurement + employment × measurement + mean hearing threshold × measurement + tinnitus onset associated with stress × measurement + tinnitus type × measurement + tinnitus influenced by stress × measurement + hearing aids × measurement + hyperacusis × measurement + number of traumatic experiences × measurement + SOMS baseline × measurement + STAI baseline × measurement + TQ baseline × measurement + HADS anxiety baseline × measurement + HADS depression baseline × measurement + SF-12 physical component summary baseline × measurement + SF-12 mental component summary baseline × measurement + hair-cortisol baseline × measurement + hair-BDNF baseline × measurement + (1 | subject)”. Reduced model: “PSQ-20 ~ measurement + age + traumatic experiences + HADS anxiety baseline + HADS depression baseline + SF-12 mental component summary baseline + age × measurement + (1 | subject)”. BDNF, Brain-Derived Neurotrophic Factor; HADS, Hospital Anxiety and Depression Scale; PDS, Posttraumatic Diagnostic Scale; PSQ-20, Perceived Stress Questionnaire (20 item version); SF-12, Short Form-12 Health Survey; SOMS, Screening of Somatoform Disorders; STAI, State-Trait Anxiety Inventory (State Anxiety); TQ, Tinnitus Questionnaire*.

* *p < 0.05*;

** *p < 0.01*;

*** *p < 0.001*.

#### Hair-Cortisol: No Change Across Baseline and Follow-Up

To investigate the change in hair-cortisol levels (research question 2) and relevant modulating influences (research question 3), two linear mixed-effects models were calculated, the first including all potentially relevant factors (full model) and the second after excluding non-significant factors by backward elimination (reduced model); the results of both models can be found in Table 5. After adjustment for multiple testing, no effect in the reduced model remained significant.

**Table 5:** Full and backward reduced linear mixed-effects models for change in hair-cortisol levels across baseline and follow-up (*N* = 80).

	**Full model**	**Backward reduced model**
	**Fixed effects estimates (95% confidence intervals**)	
Measurement	−0.14 (−0.39, 0.10)	
Sex: male (vs. female)	0.02 (−0.31, 0.34)	
Age	0.13 (−0.06, 0.32)	
Cohabiting / married (vs. single)	−0.04 (−0.23, 0.16)	
Separated / divorced / widowed (vs. single)	−0.05 (−0.31, 0.20)	
Education: linear	−0.11 (−0.41, 0.20)	
Education: quadratic	−0.01 (−0.25, 0.24)	
Employment: no (vs. yes)	0.08 (−0.10, 0.26)	
Mean hearing threshold	−0.16 (−0.41, 0.09)	
Matched tinnitus frequency	0.01 (−0.15, 0.18)	
Matched tinnitus loudness	0.08 (−0.16, 0.32)	
Tinnitus onset associated with stress: yes (vs. no)	−0.16 (−0.46, 0.15)	
Constant tinnitus (vs. intermittent)	0.04 (−0.08, 0.16)	
Tinnitus influenced by stress: yes (vs. no)	0.14 (−0.03, 0.32)	
Hearing aids: yes (vs. no)	0.004 (−0.19, 0.19)	
Hyperacusis: yes (vs. no)	0.09 (−0.06, 0.24)	
Number of traumatic experiences	0.05 (−0.11, 0.21)	
SOMS baseline	−0.02 (−0.20, 0.17)	
STAI baseline	0.21 (−0.02, 0.44)	
TQ baseline	−0.06 (−0.24, 0.13)	
PSQ-20 baseline	−0.13 (−0.41, 0.15)	
HADS anxiety baseline	−0.11 (−0.34, 0.13)	
HADS depression baseline	−0.10 (−0.35, 0.15)	−0.12 (−0.21, −0.02)
SF-12 physical component summary baseline	−0.05 (−0.26, 0.16)	
SF-12 mental component summary baseline	−0.12 (−0.38, 0.14)	−0.11 (−0.20, −0.01)
Sampling: spring (vs. winter)	0.05 (−0.08, 0.18)	
Sampling: summer (vs. winter)	0.17 (0.02, 0.31)	
Sampling: autumn (vs. winter)	0.06 (−0.10, 0.23)	
BMI	0.03 (−0.04, 0.10)	
Alcohol units per week	−0.02 (−0.09, 0.05)	
Regular use of hair products	−0.03 (−0.32, 0.26)	
Smoking: yes (vs. no)	−0.28 (−0.54, −0.01)	
Hair color: blonde / red (vs. gray / white)	0.10 (−0.31, 0.52)	
Hair color: brown / black (vs. gray / white)	−0.09 (−0.50, 0.32)	
Measurement × sex	0.10 (−0.08, 0.28)	
Measurement × age	−0.08 (−0.19, 0.02)	
Measurement × education (linear)	−0.06 (−0.23, 0.11)	
Measurement × education (quadratic)	0.04 (−0.10, 0.17)	
Measurement × mean hearing threshold	0.02 (−0.11, 0.15)	
Measurement × matched tinnitus frequency	−0.01 (−0.10, 0.09)	
Measurement × matched tinnitus loudness	0.03 (−0.10, 0.17)	
Measurement × tinnitus onset associated with stress	0.06 (−0.11, 0.23)	
Measurement × number of traumatic experiences	−0.03 (−0.11, 0.06)	
Measurement × SOMS baseline	−0.03 (−0.13, 0.07)	
Measurement × STAI baseline	−0.10 (−0.22, 0.03)	
Measurement × TQ baseline	0.09 (−0.01, 0.18)	
Measurement × PSQ-20 baseline	0.04 (−0.11, 0.20)	
Measurement × HADS anxiety baseline	0.06 (−0.07, 0.19)	
Measurement × HADS depression baseline	−0.03 (−0.17, 0.11)	
Measurement × SF-12 physical component summary baseline	0.08 (−0.04, 0.19)	
Measurement × SF-12 mental component summary baseline	−0.02 (−0.17, 0.12)	
Measurement × regular use of hair products	0.03 (−0.13, 0.19)	
Measurement × hair color: blonde / red (vs. gray / white)	−0.06 (−0.29, 0.16)	
Measurement × hair color: brown / black (vs. gray / white)	0.06 (−0.17, 0.28)	
Constant	−1.42^***^ (−1.89, −0.96)	−1.44^***^ (−1.50, −1.37)
	**Random effects variance (SD)**	
Subject (random intercept)	0.06 (0.25)	0.06 (0.24)
	**Model fit**	
Log-likelihood	−111.82	−36.20
Aikake information criterion	337.64	82.39
Bayesian information criterion	512.92	97.77
Marginal R^2^	0.27	0.06
Conditional R^2^	0.69	0.59

*Linear mixed model fit by REML; z-tests were used to test fixed effects estimates; significance levels are displayed after adjustment for multiple testing with Holm's method. Observations = 160. **Model equations**: Full model: “Hair-cortisol ~ measurement + sex × measurement + age × measurement + marital status + education × measurement + employment + mean hearing threshold × measurement + matched tinnitus frequency × measurement + matched tinnitus loudness × measurement + tinnitus onset associated with stress × measurement + tinnitus type + tinnitus influenced by stress + hearing aids + hyperacusis + number of traumatic experiences × measurement + SOMS baseline × measurement + STAI baseline × measurement + TQ baseline × measurement + PSQ-20 baseline × measurement + HADS anxiety baseline × measurement + HADS depression baseline × measurement + SF-12 physical component summary baseline × measurement + SF-12 mental component summary baseline × measurement + season of sample collection + BMI + alcohol units per week + regular use of hair products × measurement + smoking + hair color × measurement + (1 | subject).” Reduced model: “Hair-cortisol ~ HADS depression baseline + SF-12 mental component summary baseline + (1 | subject)”. BDNF, Brain-Derived Neurotrophic Factor; BMI, Body Mass Index; HADS, Hospital Anxiety and Depression Scale; PDS, Posttraumatic Diagnostic Scale; PSQ-20, Perceived Stress Questionnaire (20 item version); SF-12, Short Form-12 Health Survey; SOMS, Screening of Somatoform Disorders; STAI, State-Trait Anxiety Inventory (State Anxiety); TQ, Tinnitus Questionnaire*.

*** *p < 0.001*.

#### Hair-BDNF: No Change Across Baseline and Follow-Up

To investigate the change in hair-BDNF levels (research question 2) and relevant modulating influences (research question 3), two linear mixed-effects models were calculated, the first including all potentially relevant factors (full model) and the second after excluding non-significant factors by backward elimination (reduced model); the results of both models can be found in Table 6. The following significant fixed effects estimates were identified in the reduced model after adjustment for multiple testing (see Figure 4): Generally higher hair-BDNF levels in patients with a higher mean hearing threshold, β = 10.79 [3.64, 17.93], *z* = 2.96, *p*_unadjusted_ = 0.003, *p*_adjusted_ = 0.049, generally lower hair-BDNF levels in patients with higher tinnitus loudness, β = −11.59 [−18.98, −4.19], *z* = −3.07, *p*_unadjusted_ = 0.002, *p*_adjusted_ = 0.040, and generally lower hair-BDNF levels in patients with higher TQ baseline scores, β = −9.58 [−14.21, −4.96], *z* = −4.06, *p*_unadjusted_ < 0.001, *p*_adjusted_ = 0.001. No significant change in hair-BDNF levels across measurements was present.

**Table 6:** Full and backward reduced linear mixed-effects models for change in hair-BDNF levels across baseline and follow-up (*N* = 80).

	**Full model**	**Backward reduced model**
	**Fixed effects estimates (95% confidence intervals)**	
Measurement	2.60 (−19.37, 24.58)	0.18 (−5.57, 5.93)
Sex: male (vs. female)	−11.60 (−40.72, 17.53)	
Age	−0.59 (−17.57, 16.39)	
Cohabiting / married (vs. single)	−3.88 (−21.91, 14.16)	
Separated / divorced / widowed (vs. single)	4.24 (−19.59, 28.07)	
Education: linear	−5.05 (−32.09, 21.98)	
Education: quadratic	−6.17 (−29.04, 16.69)	
Employment: no (vs. yes)	6.89 (−8.63, 22.40)	
**Mean hearing threshold**	−11.28 (−33.08, 10.53)	10.79^*^(3.64, 17.93)
Matched tinnitus frequency	−8.16 (−23.02, 6.70)	
**Matched tinnitus loudness**	12.07 (−10.12, 34.26)	−11.59^*^(−18.98, **−4.19)**
Tinnitus onset associated with stress: yes (vs. no)	4.18 (−22.82, 31.19)	
Constant tinnitus (vs. intermittent)	−7.00 (−17.32, 3.31)	
Tinnitus influenced by stress: yes (vs. no)	−3.15 (−18.55, 12.26)	
Hearing aids: yes (vs. no)	11.74 (−5.05, 28.53)	
Hyperacusis: yes (vs. no)	−5.74 (−19.22, 7.74)	
Number of traumatic experiences	−18.61 (−32.45, −4.77)	−13.13 (−23.00, −3.26)
SOMS baseline	1.38 (−15.57, 18.33)	
STAI baseline	6.22 (−13.86, 26.29)	
**TQ baseline**	−12.20 (−28.20, 3.80)	−9.58^**^(−14.21, **−4.96)**
PSQ-20 baseline	9.44 (−15.02, 33.90)	
HADS anxiety baseline	−11.30 (−32.34, 9.74)	
HADS depression baseline	10.71 (−10.99, 32.41)	
SF-12 physical component summary baseline	−4.52 (−23.24, 14.19)	
SF-12 mental component summary baseline	13.84 (−9.17, 36.84)	
Shift work: yes (vs. no)	43.59 (6.29, 80.88)	16.44 (4.35, 28.53)
Sport: linear	9.22 (−8.38, 26.82)	
Sport: quadratic	7.51 (−4.26, 19.29)	
Sport: cubic	3.56 (−5.82, 12.94)	
Hair color: blonde / red (vs. gray / white)	−7.55 (−44.32, 29.23)	
Hair color: brown / black (vs. gray / white)	−5.39 (−42.42, 31.64)	
Frequency of hair washing per week	3.90 (−9.28, 17.09)	
Sampling: spring (vs. winter)	−4.76 (−16.33, 6.80)	
Sampling: summer (vs. winter)	−3.81 (−16.38, 8.76)	
Sampling: autumn (vs. winter)	−9.57 (−24.30, 5.17)	
BMI	−1.40 (−7.27, 4.47)	
Alcohol units per week	4.94 (−1.38, 11.26)	
Physical activity score	−0.73 (−6.61, 5.15)	
Regular use of hair products	12.83 (−12.41, 38.06)	
Smoking: yes (vs. no)	−13.27 (−36.00, 9.46)	
Cups of coffee / tea per day	−0.35 (−7.60, 6.90)	
Measurement × sex	4.48 (−11.84, 20.79)	
Measurement × age	−0.36 (−10.36, 9.63)	
Measurement × education (linear)	0.80 (−14.60, 16.19)	
Measurement × education (quadratic)	5.37 (−7.55, 18.30)	
Measurement × mean hearing threshold	11.18 (−0.60, 22.95)	
Measurement × matched tinnitus frequency	7.12 (−1.71, 15.96)	
Measurement × matched tinnitus loudness	−12.58 (−24.90, −0.27)	
Measurement × tinnitus onset associated with stress	1.23 (−14.11, 16.56)	
Measurement × number of traumatic experiences	10.53 (2.32, 18.73)	6.50 (0.73, 12.26)
Measurement × SOMS baseline	−2.70 (−11.90, 6.50)	
Measurement × STAI baseline	−1.60 (−13.11, 9.91)	
Measurement × TQ baseline	4.55 (−4.26, 13.35)	
Measurement × PSQ-20 baseline	−6.99 (−21.06, 7.08)	
Measurement × HADS anxiety baseline	5.77 (−6.26, 17.80)	
Measurement × HADS depression baseline	−7.89 (−20.01, 4.22)	
Measurement × SF-12 physical component summary baseline	3.06 (−7.24, 13.35)	
Measurement × SF-12 mental component summary baseline	−8.19 (−21.45, 5.06)	
Measurement × shift work	−20.80 (−41.04, −0.57)	
Measurement × hair color: blonde / red (vs. gray / white)	5.10 (−15.58, 25.77)	
Measurement × hair color: brown / black (vs. gray / white)	1.29 (−19.00, 21.58)	
Measurement × frequency of hair washing per week	−2.76 (−10.09, 4.56)	
Measurement × regular use of hair products	−5.59 (−20.06, 8.88)	
Constant	87.56^***^ (45.29, 129.84)	75.29^***^ (65.34, 85.25)
	**Random effects variance (SD)**	
Subject (random intercept)	364.91 (19.10)	251.76 (15.87)
	**Model fit**	
Log-likelihood	−548.79	−710.35
Aikake information criterion	1,229.59	1,440.70
Bayesian information criterion	1,432.55	1,471.45
Marginal R^2^	0.36	0.29
Conditional R^2^	0.69	0.59

*Linear mixed model fit by REML; z-tests were used to test fixed effects estimates; significance levels are displayed after adjustment for multiple testing with Holm's method; significant effects in the reduced model are printed in bold. Observations = 160. **Model equations:** Full model: “BDNF ~ measurement + sex × measurement + age × measurement + marital status + education × measurement + employment + mean hearing threshold × measurement + matched tinnitus frequency × measurement + matched tinnitus loudness × measurement + tinnitus onset associated with stress × measurement + tinnitus type + tinnitus influenced by stress + hearing aids + hyperacusis + number of traumatic experiences × measurement + SOMS baseline × measurement + STAI baseline × measurement + TQ baseline × measurement + PSQ-20 baseline × measurement + HADS anxiety baseline × measurement + HADS depression baseline × measurement + SF-12 physical component summary baseline × measurement + SF-12 mental component summary baseline × measurement + shift work × measurement + sport + hair color × measurement + hair washing frequency × measurement + season of sample collection + BMI + alcohol units per week + regular use of hair products × measurement + smoking + cups of coffee/tea per day + (1 | subject).” Reduced model: “BDNF ~ measurement + mean hearing threshold + matched tinnitus loudness + number of traumatic experiences + TQ baseline + shift work + number of traumatic experiences × measurement + (1 | subject)”. BDNF, Brain-Derived Neurotrophic Factor; BMI, Body Mass Index; HADS, Hospital Anxiety and Depression Scale; PDS, Posttraumatic Diagnostic Scale; PSQ-20, Perceived Stress Questionnaire (20 item version); SF-12, Short Form-12 Health Survey; SOMS, Screening of Somatoform Disorders; STAI, State-Trait Anxiety Inventory (State Anxiety); TQ, Tinnitus Questionnaire*.

* *p < 0.05*;

** *p < 0.01*;

*** *p < 0.001*.

### Exploratory Analysis: Cross-Lagged Panel Model

**Figure 5:**
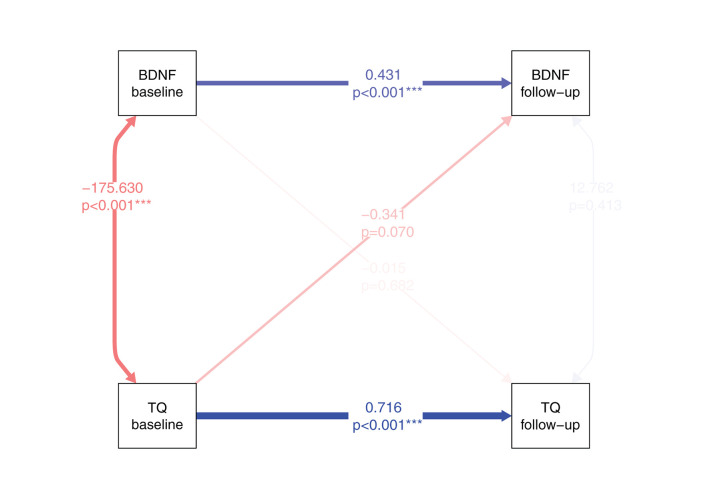
Cross-lagged panel model (structural equation model) for the temporal relations between TQ scores and hair-BDNF values from baseline to follow-up. Blue lines indicate positive and red lines negative associations; line width indicates association strength. Numbers indicate standardized estimates and respective *p*-values. For the prediction of BDNF, mean hearing threshold and tinnitus loudness were included as control variables; for the prediction of TQ, concomitant SF-12 physical component summary scores and the dummy variable “marital status: separated / divorced / widowed” were included as control variables. To simplify the figure, control variables are not depicted. Model fit: *N* = 80, χ^2^ = 36.807, df = 12, *p* < 0.001; RMSEA = 0.144. BDNF, Brain-Derived Neurotrophic Factor; TQ, Tinnitus Questionnaire; SF-12, Short Form-12 Health Survey.

For research question 4, based on the linear mixed-effects model results indicating an effect of TQ baseline scores on hair-BDNF levels across measurements, a cross-lagged panel model in a structural equation modeling framework was calculated. This model investigates the temporal relationships between TQ scores and hair-BDNF values while accounting for their stability over time and controlling for other identified influencing factors (see Figure 5).

Both TQ scores, β = 0.716, SE = 0.074, *p* < 0.001, and hair-BDNF values, β = 0.431, SE = 0.119, *p* < 0.001, were stable over the investigated 3-month period; with higher stability of TQ scores. The two measures showed significantly negative covariance at baseline, ψ = −175.630, SE = 45.969, *p* < 0.001, but not at follow-up, ψ = 12.762, SE = 15.589, *p* = 0.413. There was a trend toward statistical significance for the effect of TQ scores at baseline to predict hair-BDNF at follow-up, β = −0.341, SE = 0.188, *p* = 0.070, while the opposite cross-lagged path (of hair-BDNF at baseline to predict TQ scores at follow-up) was non-significant, β = −0.015, SE = 0.037, *p* = 0.682. Approximately 62% of the variance in TQ scores at follow-up (*R*^2^ = 0.621), and approximately 36% of the variance in hair-BDNF values at follow-up (*R*^2^ = 0.355), was accounted for by the model.

## Discussion

In summary, we found that the compact multimodal tinnitus-specific cognitive behavioral therapy effectively reduced tinnitus-related distress and perceived stress levels, in line with our hypothesis (research question 1). However, hair-cortisol and hair-BDNF levels did not reflect these improvements, contrary to our expectations (research question 2). Furthermore, the magnitude of the therapeutic effects was not influenced by the investigated factors (sociodemographic, tinnitus-/hearing-related, psychological, or biological) (research question 3), but some general associations (across all measurements) were identified. Separated, divorced, or widowed patients showed generally higher levels of tinnitus-related distress, which were, in turn, related to lower physical health-related quality of life (QoL). Higher perceived stress levels, on the other hand, were associated with higher anxiety and lower mental health-related QoL. Neither baseline hair-cortisol nor hair-BDNF levels were associated with psychological treatment outcomes, indicating that these biomarkers had no predictive clinical value in the present study. For hair-cortisol, no predictive influences were identified; for hair-BDNF, general associations with tinnitus-related distress, tinnitus loudness, and hearing threshold were found. The exploratory cross-lagged panel analysis (research question 4) tentatively suggests that the possibility of a time-lagged effect of tinnitus-related distress affecting hair-BDNF levels is more likely than the opposite effect. However, this effect was only observed as an uncorrected trend (*p* = 0.070).

A possible explanation for the absence of changes in hair-cortisol and hair-BDNF levels in the present study might be the relatively short treatment duration and follow-up period. The cognitive behavioral therapy-based multimodal treatment, which constitutes the current standard clinical treatment for chronic tinnitus offered at the Tinnitus Center (Charité – Universitätsmedizin Berlin), resulted in measurable reductions in tinnitus-related distress (−13.3%) and perceived stress (−11.5%) three months later. However, these reductions may not have been substantial enough to induce biological changes, or a longer period might have been needed to detect such changes. Regarding cortisol, Li et al. (46) examined the effects of a treatment intervention that combined cognitive behavioral therapy with masking therapy and sound treatment and lasted six months. In addition to a decrease in tinnitus-related distress, they found a decrease in serum cortisol levels in chronic tinnitus patients, suggesting that a longer treatment duration may be necessary to measurably affect the hypothalamic—pituitary—adrenal (HPA) axis function. Moreover, findings on the association between hair-cortisol and measures of perceived stress are inconsistent (18) and previous studies examining the effects of psychological interventions aimed at stress reduction on hair-cortisol levels in different highly stressed study populations made diverging findings. While similar to our results, some found decreases in perceived stress levels that were not accompanied by changes in hair-cortisol levels (47, 48), others observed reductions in hair-cortisol levels following the treatment intervention (49, 50). More research is needed to explore the relationship of hair-cortisol with stress reduction by psychological treatment interventions in different highly stressed groups and to disentangle methodological and treatment-related influences.

Measurement of BDNF in hair is a relatively new method first used in a pilot study by Harb et al. (19). In this study, it was shown that BDNF can be measured in hair samples using a commercially available BDNF assay, that hair-BDNF negatively correlates with hair-cortisol, is associated with hair-biology measures indicative of stress-induced dyshomeostasis, and is a stable measure across independent samples. While immunohistology of human hair follicles confirms BDNF incorporation into hair (51), additional validation studies for the quantification of hair-BDNF are needed. However, the good intra- and inter-assay coefficients of variation observed here indicate a sound methodological approach.

Although we observed no treatment effect for hair-BDNF, general associations of baseline tinnitus-related distress, tinnitus loudness, and hearing threshold with hair-BDNF levels at both measurements were found, extending our cross-sectional findings (baseline measurements) in the same sample (28). Louder tinnitus was related to lower hair-BDNF and higher hearing thresholds to higher hair-BDNF levels at baseline and follow-up. However, the previously observed positive cross-sectional association between hearing aid use and hair-BDNF levels (at baseline) was not observed here, possibly due to the higher number of hair samples included in the present longitudinal analysis. While the negative effect of tinnitus loudness might reflect detrimental distress-related influences on neuroplasticity, the positive effect of mean hearing threshold was surprising. However, the relationship between hearing loss and neuroplasticity is complex. Neuroanatomical studies found that hearing loss in older adults is associated with volume decreases of the primary auditory cortex (52, 53). However, in middle-aged hearing-impaired subjects, volume increases in the auditory association cortex (Brodmann area 22) have been observed (54), as well as volume increases of the angular gyrus (55); both findings are likely indicative of compensatory mechanisms (54, 55). As most of our participants were middle-aged and had mostly no-to-mild hearing impairment, the observed association might potentially be related to compensatory neuroplasticity alterations in certain brain regions and associated increased BDNF levels. However, this explanation is entirely speculative and requires further investigation.

Regarding BDNF measured in blood, evidence shows that serum/plasma BDNF levels increase in response to antidepressant treatment in patients with major depressive disorder (24, 26, 56). The magnitude of the respective change in BDNF levels appears to be positively related to treatment duration (24). Similarly, peripheral BDNF levels were found to increase after several weeks of mindfulness-based interventions (27). Compared with the literature, it seems likely that the treatment duration of 5 days in the present study, even though leading to relevant psychological changes, may have been too short to induce BDNF changes. Moreover, in contrast to our results, Xiong et al. (57) observed a decrease in plasma BDNF levels in patients with severe tinnitus three months after tinnitus retraining therapy (counseling and sound therapy). However, they found no correlation between plasma BDNF and tinnitus severity or loudness, which is also contrary to our results. Differences between Xiong et al. (57) and the present study include sample characteristics (*N* = 14 with severe tinnitus vs. *N* = 80 with predominantly moderate tinnitus), treatment approach (3-month tinnitus retraining therapy vs. 5-day compact multimodal tinnitus-specific cognitive behavioral therapy), sampling type (blood vs. hair), and methodological differences, all of which may have influenced the conflicting results.

Despite the absence of treatment-induced changes in hair-BDNF levels, our exploratory results tentatively suggest the possibility of a time-lagged effect of tinnitus-related distress (at baseline) affecting hair-BDNF levels (at follow-up). While this trend needs to be tested in larger-scale studies, it may further indicate that more substantial treatment-induced changes in tinnitus-related distress may be necessary to elicit measurable changes in hair-BDNF levels. Overall, further research is needed for a better understanding of the relationship between tinnitus-related distress and hair-BDNF levels.

In addition to treatment duration and follow-up period, other factors might have influenced the observed lack of changes in hair-cortisol and hair-BDNF values. Even though many covariates with potential associations to the investigated biomarkers were included (sociodemographic, psychological, tinnitus-/hearing-related, lifestyle, and hair-related), not all potentially confounding factors could be controlled for, e.g., medical comorbidities and medication. However, none of the participants suffered from endocrine diseases with altered cortisol production or neurodegenerative diseases (Alzheimer's, Parkinson's, or Huntington's disease) with known changes in cortisol and BDNF levels (44, 45). Moreover, confounding influences of antidepressant medication appear unlikely, as biomarker levels did not significantly differ between participants taking antidepressants and those not taking antidepressants (although there was a trend observed for hair-cortisol). However, influences of other medical comorbidities or medications might have been present.

Musculoskeletal symptoms (muscular imbalance, segmental joint dysfunction, chronic cervical syndrome, craniomandibular/temporomandibular dysfunction, and bruxism) were common in our sample (39–58%). While we did not specifically assess the presence of somatosensory tinnitus; i.e., tinnitus which is influenced by somatosensory afference from the cervical spine or temporomandibular area (58), the relatively high frequency of the reported musculoskeletal symptoms suggests that for a subgroup in our sample, somatosensory influences on tinnitus might have been present. Regarding somatosensory tinnitus, cervical muscle tension, particularly in upper posterior muscle groups, might in some cases have a pathophysiological role in tinnitus – likely in combination with stress (59).

While physical and mental symptoms appear generally interlinked in bothersome tinnitus (60), the interplay between stress, muscle tension, and tinnitus burden appears especially important for tinnitus with somatosensory influences. The multimodal treatment in this study also included physiotherapeutic elements. Therefore, beneficial treatment effects on the described musculoskeletal symptoms might have been present, although we did not investigate them. Consequently, in the subgroup of patients with somatosensory tinnitus, the treatment might have contributed to the improvement of tinnitus-related distress via reducing muscular tension. Overall, further research is needed for a better understanding of stress-related pathophysiological and therapeutic effects in chronic tinnitus.

### Limitations

There are several limitations to this study. First, as no control group was included, the observed treatment effects cannot be clearly distinguished from other time effects, e.g., natural habituation over time. Moreover, no information was collected regarding more long-term effects after the 3-month follow-up measurement. In addition, the significance level was adjusted for multiple testing for the main analysis; however, the exploratory cross-lagged panel analysis faces potential validity limitations. Aspects of the treatment delivery and study design may have influenced the results and thus limit their generalizability. Insufficient power in our study might be an explanation for the lack of treatment effects in the assessed hair-biomarkers. However, the width of the confidence interval around the null effect of change in hair-BDNF levels was similar to that of the observed significant effects on hair-BDNF levels (reduced model), thus suggesting reasonable accuracy in the estimation. For hair-cortisol, on the other hand, the measurement variable was not selected to be included in the reduced model, and no significant effects were observed, which might indicate greater uncertainty in the estimation. Additional explanations for the lack of treatment effects might be potential confounding influences, e.g., by medical comorbidities or medication. Overall, the non-significant biomarker results need to be interpreted with caution. In addition, some follow-up measurements were performed during the beginning of the COVID-19 pandemic in Germany (*N* = 9 after March 2020), which might have affected the stress level of these participants.

## Conclusion

Three months after compact multimodal tinnitus-specific cognitive behavioral therapy lasting for 5 days, reductions in tinnitus-related distress and perceived stress were observed. This suggests that the treatment (consisting of cognitive behavioral therapy, education, counseling, otorhinolaryngological and general medical diagnostics, auditory attention training, relaxation, and physiotherapeutic sessions) was successful in reducing tinnitus burden beyond the clinical setting in patients' daily lives. Generally, higher tinnitus-related distress was related to being separated, divorced, or widowed and to lower physical health-related QoL; higher perceived stress was related to higher anxiety levels and lower mental health-related QoL. No change occurred in hair-cortisol and hair-BDNF levels and no predictive influence of baseline biomarker scores on psychometric treatment outcomes was present. For hair-cortisol, no influencing factor could be identified; for hair-BDNF, relationships with hearing threshold, tinnitus loudness, and tinnitus-related distress appear relevant. In addition, the exploratory analysis provided tentative and limited evidence of a time-lagged effect of tinnitus-related distress (at baseline) on hair-BDNF levels (at follow-up). Possible explanations for the lack of treatment effects in hair-biomarkers are the short treatment duration (5 days) and follow-up interval (12 weeks) and potential confounding by medical factors. Further studies are needed to investigate treatment-induced changes in hair-biomarkers in chronic tinnitus, especially hair-BDNF, to obtain a better understanding of stress-related effects in chronic tinnitus.

## Data Availability Statement

The datasets presented in this article are not readily available because no consent of the participants to publish their data was obtained. Requests to access the datasets should be directed to Birgit Mazurek (birgit.mazurek@charite.de).

## Ethics Statement

The studies involving human participants were reviewed and approved by the local ethic commission of Charité – Universitätsmedizin Berlin (No. EA1/035/16). The patients/participants provided their written informed consent to participate in this study.

## Author Contributions

LB: project administration, investigation, formal analysis, visualization, and writing—original draft preparation. BB: supervision and writing—reviewing and editing. PN: methodology, supervision, and writing—reviewing and editing. PB: writing—reviewing and editing. BM: conceptualization, funding acquisition, project administration, resources, supervision, and writing—reviewing and editing. EP: conceptualization, resources, project administration, and writing—reviewing and editing.

## Funding

This project has received funding from the European Union's Horizon 2020 research and innovation programme under the Marie Skłodowska-Curie Grant Agreement No 764604, and the Heinz und Heide Dürr Stiftung. We acknowledge support from the German Research Foundation (DFG) and the Open Access Publication Fund of Charité – Universitätsmedizin Berlin.

## Conflict of Interest

The authors declare that the research was conducted in the absence of any commercial or financial relationships that could be construed as a potential conflict of interest.

## Publisher's Note

All claims expressed in this article are solely those of the authors and do not necessarily represent those of their affiliated organizations, or those of the publisher, the editors and the reviewers. Any product that may be evaluated in this article, or claim that may be made by its manufacturer, is not guaranteed or endorsed by the publisher.
