# Supplementary material for: Psychological Treatment Effects Unrelated to Hair-Cortisol and Hair-BDNF Levels in Chronic Tinnitus
Source: Front Psychiatry. 2022 Feb 18;13:764368. doi: 10.3389/fpsyt.2022.764368 (PMC8895295; doi:10.3389/fpsyt.2022.764368)
Supplement: Supplementary file 1 [file Data_Sheet_1.pdf]

Supplementary Material

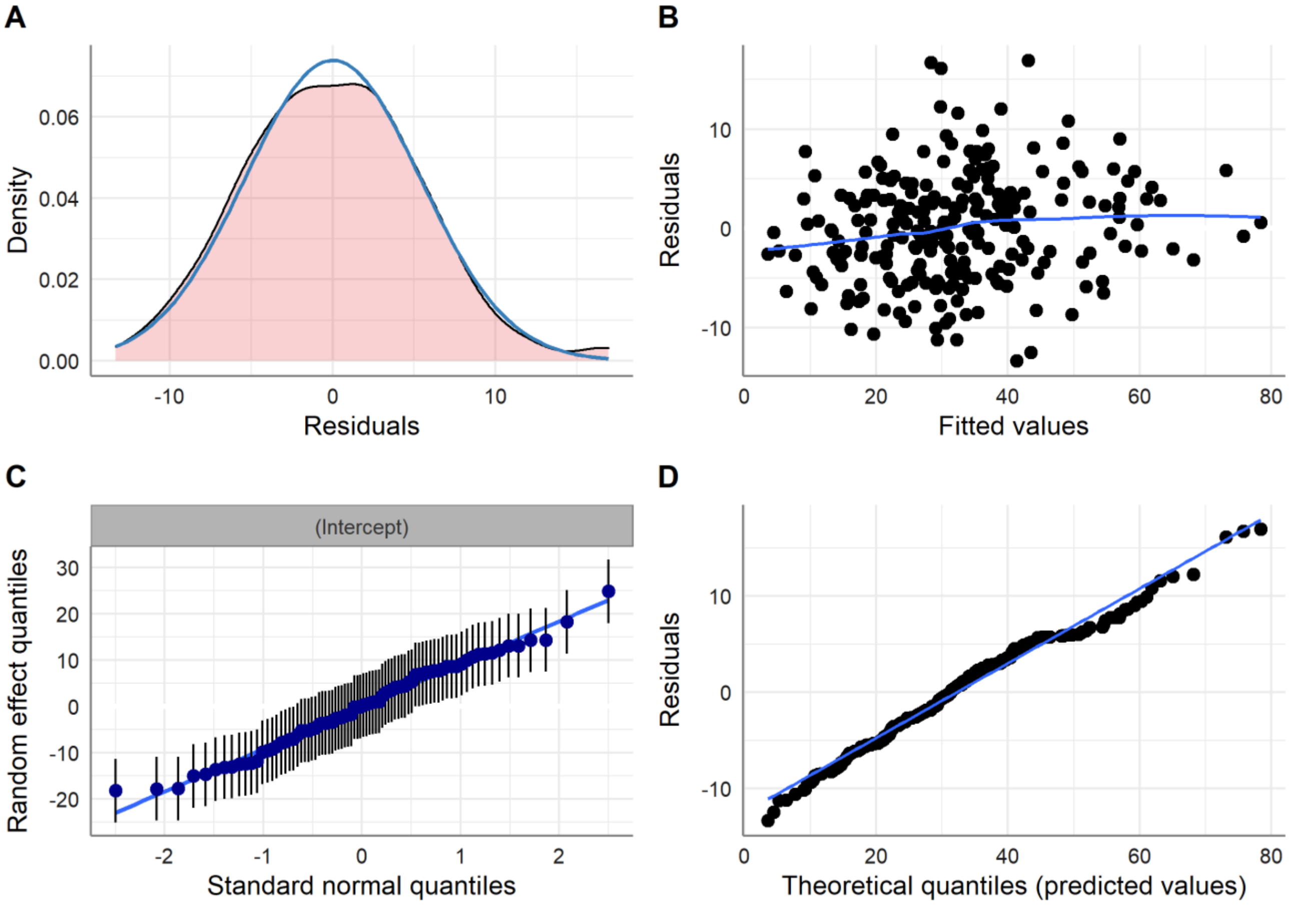

Supplementary Figure 1. Diagnostic plots for the model prediction (reduced model) of Tinnitus Questionnaire (TQ).

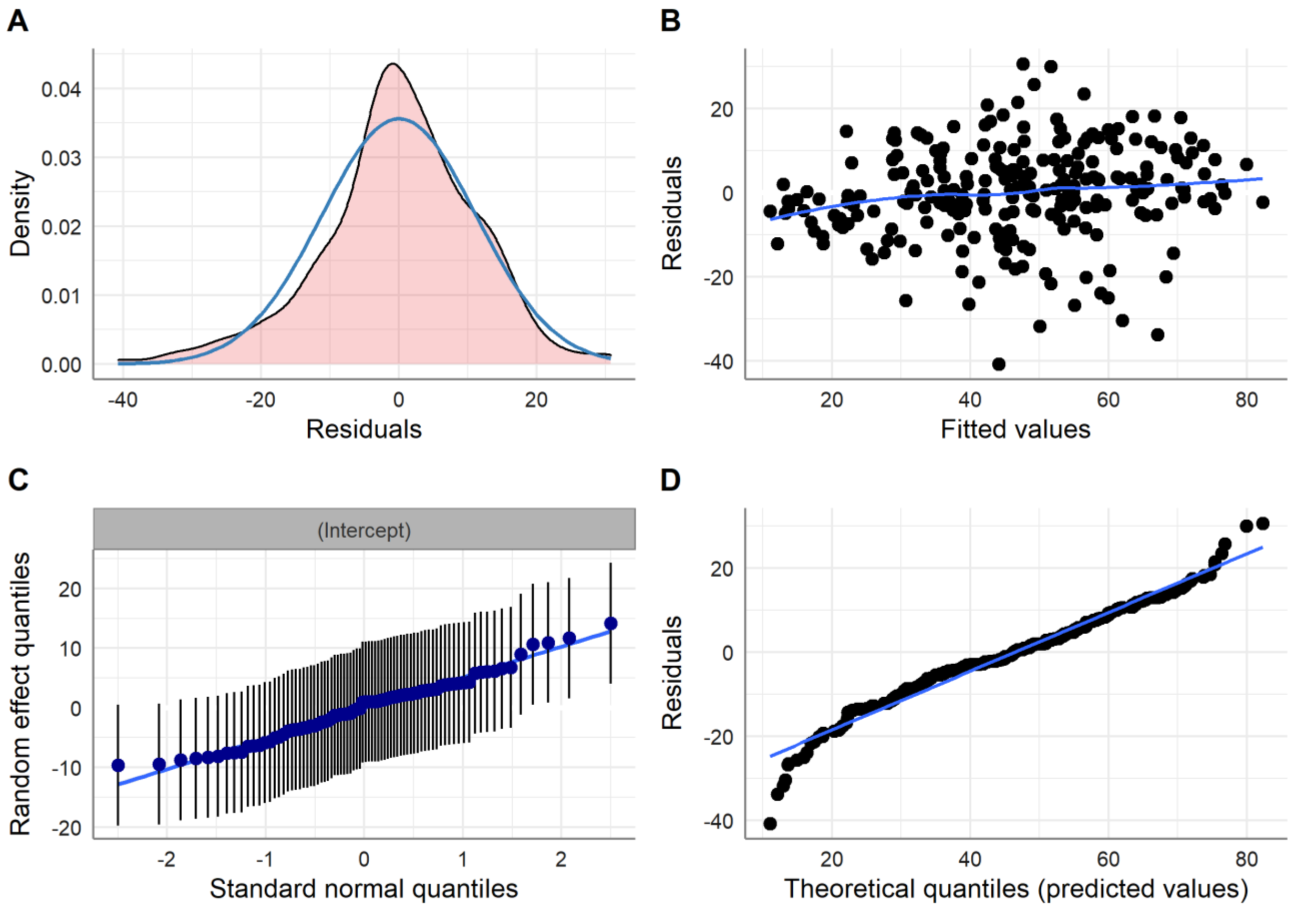

Supplementary Figure 2. Diagnostic plots for the model prediction (reduced model) of Perceived Stress Questionnaire (PSQ-20).

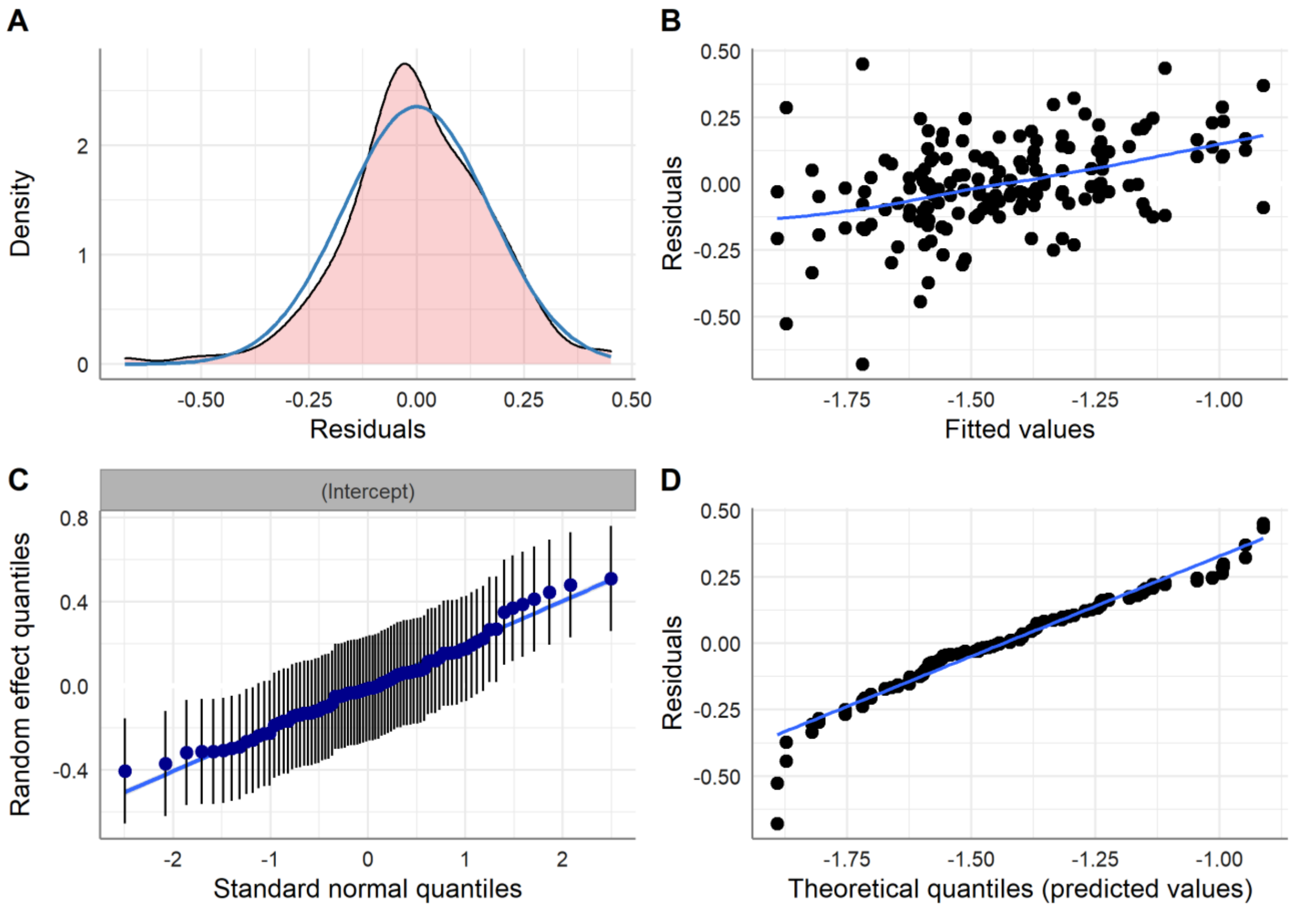

Supplementary Figure 3. Diagnostic plots for the model prediction (reduced model) of hair-cortisol.

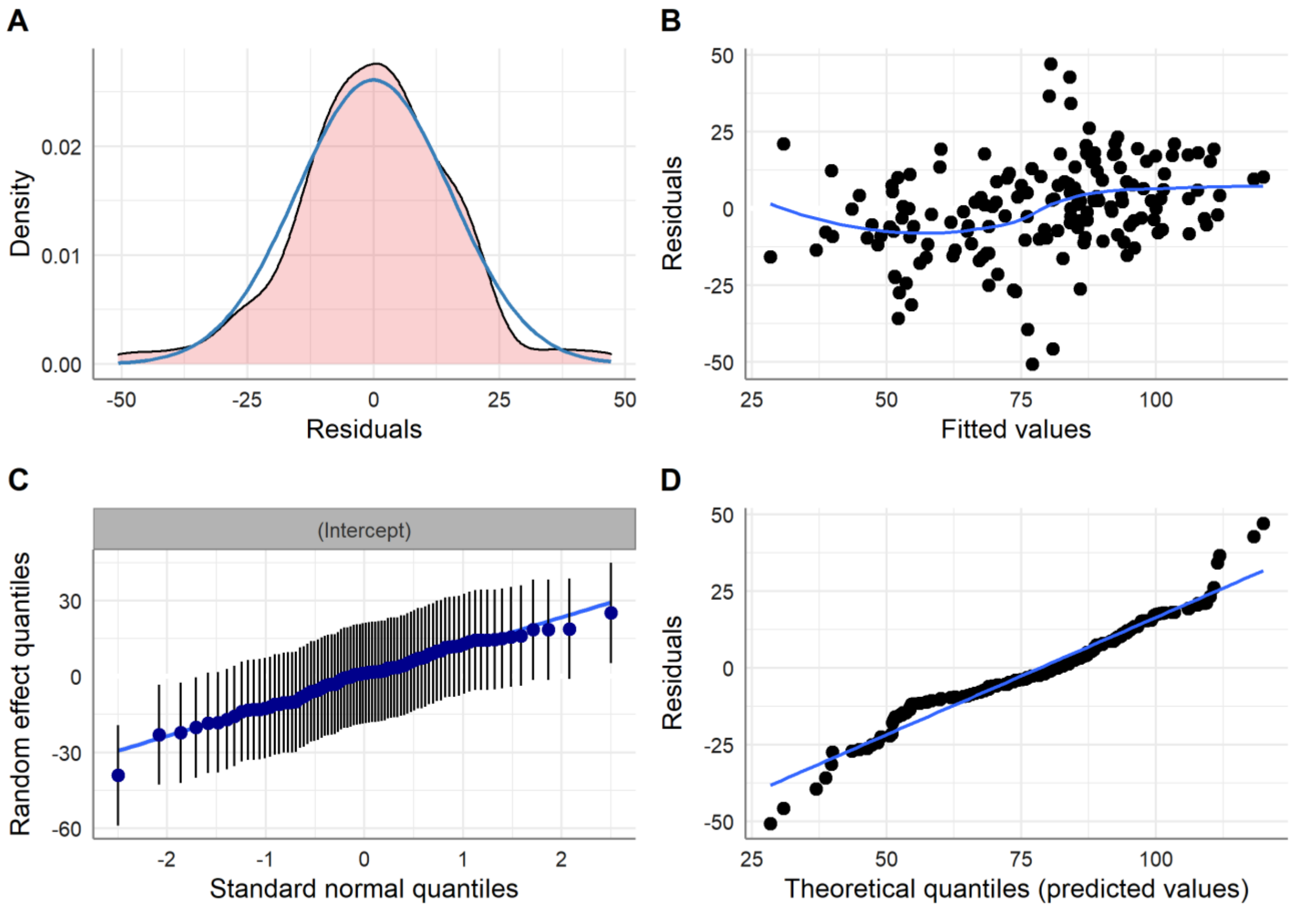

Supplementary Figure 4. Diagnostic plots for the model prediction (reduced model) of hair-BDNF.

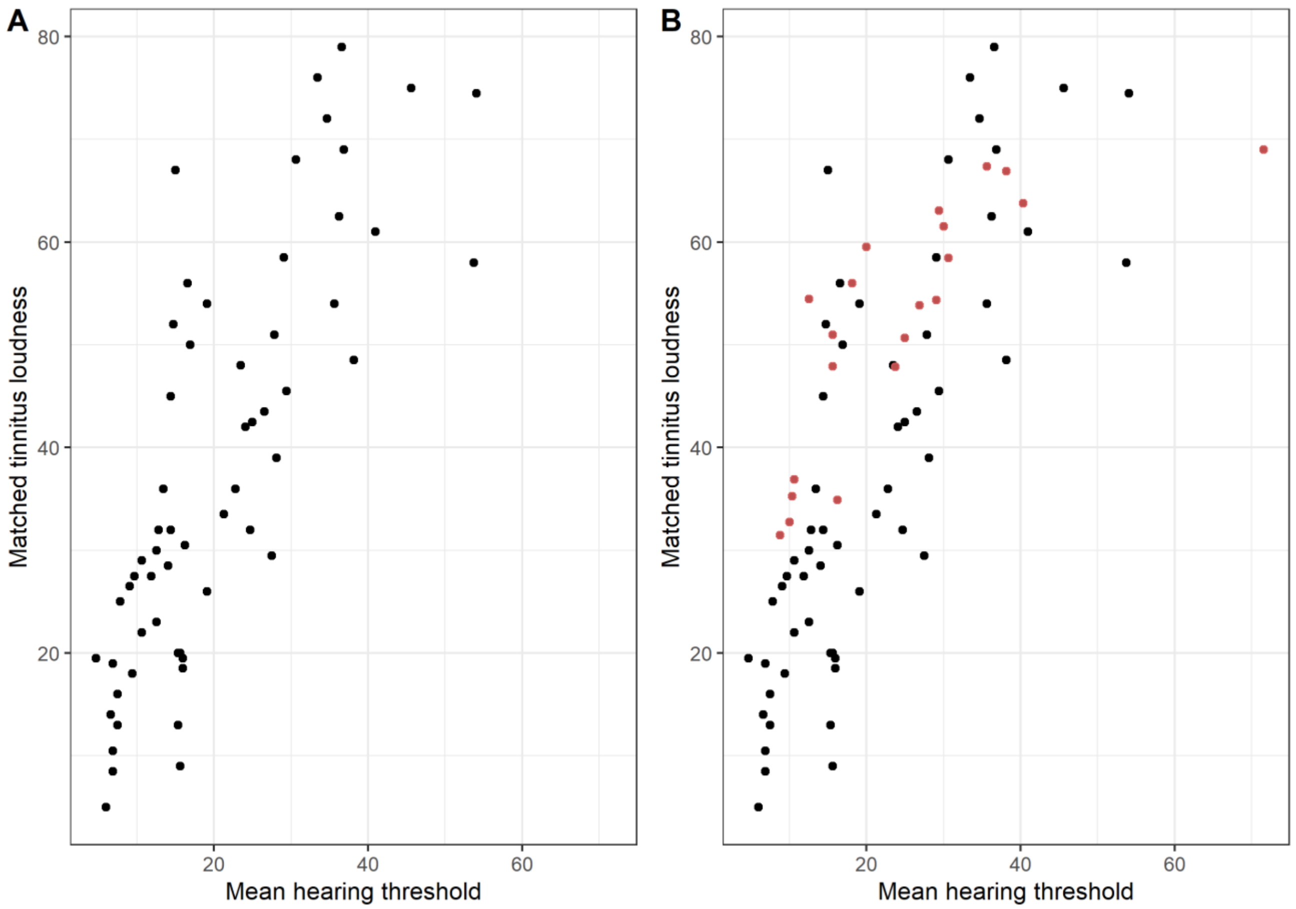

Supplementary Figure 5. Scatterplot for matched tinnitus loudness and mean hearing threshold without (A) and with (B) imputed matching data by k-nearest neighbor imputation. Values with imputed tinnitus loudness (N=21) are depicted in red.
